# Hybrid of DSR-GAN and CNN for Alzheimer disease detection based on MRI images

**DOI:** 10.1038/s41598-025-94677-9

**Published:** 2025-04-13

**Authors:** Sarah Oraby, Ahmed Emran, Basel El-Saghir, Saeed Mohsen

**Affiliations:** 1 Department of Electrical Engineering, Faculty of Engineering, Al-Azhar University, Cairo, Egypt; 2 Department of Electronics and Communications Engineering, Al-Madinah Higher Institute for Engineering and Technology, Giza, 12947 Egypt; 3 Department of Artificial Intelligence Engineering, Faculty of Computer Science and Engineering, King Salman International University (KSIU), South Sinai, 46511 Egypt

**Keywords:** Generative adversarial network (GAN), Convolutional neural network, Medical image classification, Magnetic resonance imaging (MRI), Alzheimer, Computational science, Computer science

## Abstract

In this paper, we propose a deep super-resolution generative adversarial network (DSR-GAN) combined with a convolutional neural network (CNN) model designed to classify four stages of Alzheimer’s disease (AD): Mild Dementia (MD), Moderate Dementia (MOD), Non-Demented (ND), and Very Mild Dementia (VMD). The proposed DSR-GAN is implemented using a PyTorch library and uses a dataset of 6,400 MRI images. A super-resolution (SR) technique is applied to enhance the clarity and detail of the images, allowing the DSR-GAN to refine particular image features. The CNN model undergoes hyperparameter optimization and incorporates data augmentation strategies to maximize its efficiency. The normalized error matrix and area under ROC curve are used experimentally to evaluate the CNN’s performance which achieved a testing accuracy of 99.22%, an area under the ROC curve of 100%, and an error rate of 0.0516. Also, the performance of the DSR-GAN is assessed using three different metrics: structural similarity index measure (SSIM), peak signal-to-noise ratio (PSNR), and multi-scale structural similarity index measure (MS-SSIM). The achieved SSIM score of 0.847, while the PSNR and MS-SSIM percentage are 29.30 dB and 96.39%, respectively. The combination of the DSR-GAN and CNN models provides a rapid and precise method to distinguish between various stages of Alzheimer’s disease, potentially aiding professionals in the screening of AD cases

## Introduction

**Fig. 1:**
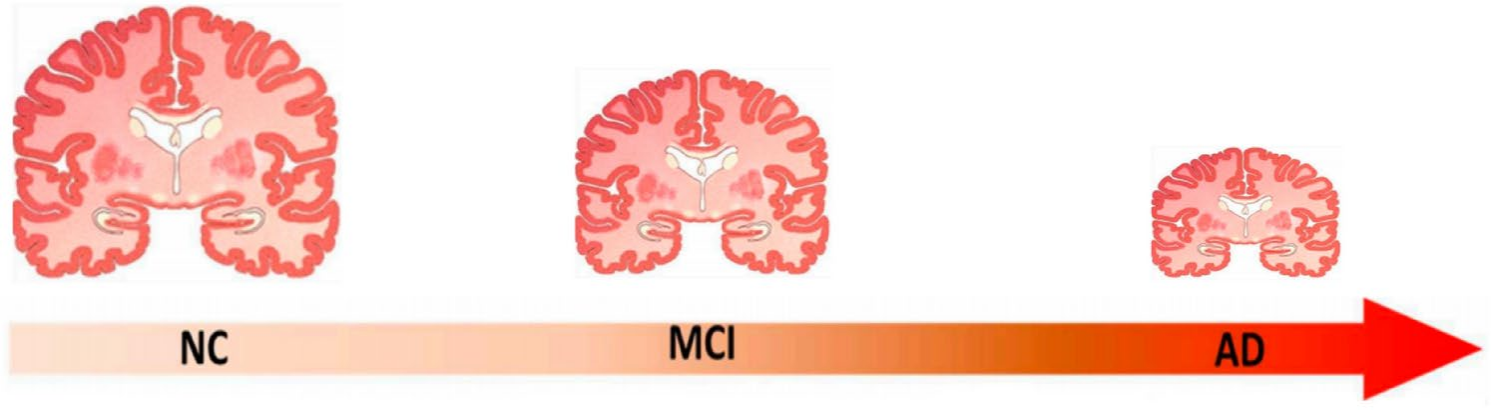
The progress of Alzheimer’s disease in brain^18^.

Alzheimer’s disease (AD) poses a significant challenge for societies around the globe. This progressive neurological disorder is primarily characterized by memory impairment. As the population ages, the incidence of Alzheimer’s disease is anticipated to rise dramatically. According to statistics from the World Alzheimer’s Report, this issue is of paramount importance. Currently, over 55 million individuals have been diagnosed with Alzheimer’s, and projections suggest that this figure could escalate to 78 million by 2030^1^. Classifying AD is essential for guiding appropriate treatment strategies^2^. Computer-aided diagnostic systems facilitate rapid diagnoses, assisting healthcare providers in their decision-making processes^3^. MRI is used for AD classification due to its superior image quality^4^. In recent years, deep learning (DL) techniques are applied in several different applications such as cardiovascular risk prediction^5^, intusion detection^6^, COVID-19 Diagnosis^7,8^, long bone fracture diagnosis^9^, and gastrointestinal cancer prediction^10^, . Also, DL techniques particularly CNNs, have been investigated for their potential in identifying Alzheimer’s disease. High-performing DL models that offer high accuracy are crucial for efficient and precise diagnoses. While existing machine learning (ML) and DL methods are employed for the diagnosis and detection of Alzheimer’s ^11^, they often suffer from low accuracy, presenting a significant obstacle^12^. Deep Super-Resolution Generative Adversarial Networks (DSR-GANs) and CNNs have emerged as promising solutions to enhance accuracy. However, these networks typically require extended training periods. GANs and CNNs have demonstrated success in a variety of fields, including the classification of AD^13,14^, human activity recognition^15^, language modeling^16^, and image processing^17^. In particular, the GAN model is advantageous as it enhances specific features of MRI images. Nevertheless, achieving sufficient precision in the detection of AD remains a critical concern. Figure 1 shows the progress of Alzheimer’s disease in brain^18^. In this Figure, it is clear that a noticeable difference in size for each type of dementia leads to a reduction in brain mass. There are three types: normal control (NC), mild cognitive impairment (MCI), and Alzheimer’s disease (AD).

This article presents a hybrid of the DSR-GAN and CNN models, the DSR-GAN consists of two components: a generator and a discriminator, which together offer architectural complexity that can refine particular features of images, improving their clarity and detail. Regarding the CNN, it is implemented to improve the accuracy of AD classification through optimized hyperparameter settings. The DSR-GAN and CNN models are trained using a dataset obtained from the Kaggle platform. Additionally, we overcomed the limited number of available MRI images through a technique called data augmentation was employed to enhance the size of dataset during the training phase.

This article has five scientific contributions:

- 1.Introducing a precise DSR-GAN combined with a CNN to effectively differentiate between Mild-Demented (MD), Moderate-Demented (MOD), Non-Demented (ND), and Very-Mild-Demented (VMD) Alzheimer’s images, enabling rapid and automated diagnoses.
- 2.Incorporating a super-resolution (SR) approach within the DSR-GAN model, leveraging GAN architecture to enhance the quality of MRI images.
- 3.Assessment of the efficiency of the DSR-GAN model through various measurements to analyze the resolution quality of the MRI images used. Additionally, evaluating of the performance of the CNN model using several metrics.
- 4.Utilizing CNN while fine-tuning its hyper-parameters to achieve enhanced accuracy, leveraging the capabilities of a graphics processing unit.
- 5.Comparing the proposed work against the latest state-of-the-art (SOTA) techniques that used for MRI image classification toward AD.

The structure of this article is organized as follows: Section "Related work" provides an overview of recent literature. Section "The proposed methodology" details the methodology employed in the proposed work. Section "Experimental results" outlines the experimental findings. Section "Discussion" offers an analysis of the results. Finally, Section "Conclusion and future work" presents the conclusion and future work of the article.

## Related work

Alzheimer’s disease, a progressive brain disorder that causes memory loss and dementia, requires early detection for better management, especially in elderly patients. The proposed system in^19^ used transfer learning with MRI data to classify Alzheimer’s into four stages—ND, VMD, MD, and MOD—achieving an accuracy of 91.7%. By utilizing AlexNet and transfer learning, the system was efficient and handled small datasets well. Future improvements included fine-tuning convolutional layers, exploring other CNN architectures, and testing with larger datasets like the Alzheimer’s Disease Neuroimaging Initiative (ADNI) dataset to aim for enhanced accuracy through both supervised and unsupervised learning methods.

Alzheimer’s disease, an incurable neurological disorder, required early detection for symptom management, yet traditional diagnostic methods were time-consuming and relied on manual feature extraction. The study in^20^ proposed an efficient ensemble model using VGG-16 and EfficientNet-B2 with transfer learning and adaptive synthetic oversampling to address imbalanced MRI data. The ensemble approach achieved a high accuracy of 97.35% and an AUC of 99.64% for multiclass datasets, outperforming single models. The model efficiently handled small datasets, automatically extracted features, and provided improved performance in Alzheimer’s diagnosis. Future work included testing with larger datasets for enhanced accuracy.

Alzheimer’s Disease, the leading cause of dementia, was challenging to diagnose with consistency using traditional methods, as they lacked sensitivity and precision. The authors of^21^ introduced a CNN-based model, DEMNET, to classify AD stages from MRI images. By addressing class imbalance with SMOTE, the model reached a high accuracy of 95.23%, AUC of 97%, and Cohen’s Kappa value of 0.93 on Kaggle data, outperforming prior methods. Tested on the ADNI dataset for robustness, the model achieved 84.83% accuracy, providing a reliable support tool for assessing AD severity based on dementia stages.

The authors of^22^ focused on early Alzheimer’s detection through DL, using neuroimaging to identify AD biomarkers. A CNN model was trained on ADNI dataset sMRI images, categorizing them into AD, mild cognitive impairment, and cognitively normal groups. By combining features from different CNN layers, the model achieved high accuracy with reduced parameters, outperforming existing methods. The fully automated system showed promise in extracting key patterns from brain images, providing a quick, reliable tool for AD diagnosis and potentially for other neurological conditions. Future improvements include incorporating patient history for enhanced diagnostic accuracy.

As noted in^23^, the study focused on developing an efficient, deep CNN model, ADD-Net, for diagnosing AD using MRI scans. Built with low parameter requirements, ADD-Net efficiently handled smaller datasets and addressed class imbalance through synthetic oversampling. Achieving a high precision of 98.63% and an AUC of 99.76%, ADD-Net outperformed established models like DenseNet169 and VGG19 in precision, recall, and F1-score. Class activation maps visualized disease regions, enhancing interpretability, and showing promise as an effective AD detection tool. Future improvements aim to incorporate transfer learning for enhanced performance.

According to^24^, the study focused on developing hybrid DL models, including CNN, Bidirectional Long Short-Term Memory (LSTM), and Stacked Deep Dense Neural Network (SDDNN), for the early detection of AD through text classification of clinical transcripts from Alzheimer’s patients. Using the DementiaBank dataset, these models were trained and evaluated based on randomly initialized and with Glove embeddings, achieving significant hyperparameter optimization via GridSearch. The SDDNN model, enhanced with Glove embeddings, achieved an accuracy of 93.31% and outperformed other models in AUC, precision, F1 score, and recall. The findings highlighted the potential of automated models for aiding clinical experts in the early diagnosis of AD, with suggestions for future work to improve performance on larger datasets.

As reported in^25^, the study aimed to implement a predictive model for Alzheimer’s Disease (AD) progression using LSTM networks, focusing on the temporal relationships in patient data rather than merely classifying current disease states. The LSTM model incorporated fully connected and activation layers to encode these temporal relationships, enabling predictions about future disease stages. Experimental results demonstrated that this model outperformed most existing approaches in predicting AD development and proved stable across different data sizes. Additionally, the findings highlighted a Cortical Thickness Average (TA) as a significant predictor of AD progression.

The authors of^26^ focused on developing an automatic diagnosis system for Alzheimer’s Disease (AD) using structural magnetic resonance imaging (sMRI) to enhance accuracy while addressing challenges like overfitting and data scarcity. By utilizing ensembles of CNNs for extracting several features and applying a patch-based approach, the researchers aimed to analyze the right and left hippocampus—key biomarkers for AD. Experiments conducted on the Alzheimer’s Disease Neuroimaging Initiative (ADNI) database yielded an accuracy of 85.55%, while testing on the National Research Center for Dementia (NRCD) dataset achieved 90.05%. The results demonstrated that this method is competitive with state-of-the-art techniques, providing an effective framework for AD diagnosis.

According to^27^, the study focused on enhancing early diagnosis of AD, which affects over 1 in 9 Americans and poses significant challenges for caregivers and healthcare systems. After reviewing existing detection approaches, the authors proposed a deep CNN architecture with 7,866,819 parameters, comprising three branches with varying kernel sizes. This model achieved a remarkable accuracy of 99.05% in classifying patients as non-demented, mildly demented, or moderately demented. The findings highlighted the potential of DL methods to enhance early AD diagnosis, facilitating timely interventions that could alleviate burdens on patients and caregivers. The study also suggested future directions, such as refining preprocessing methods and exploring multi-modal approaches for enhanced diagnostic performance.

As noted in^28^, the study focused on improving early diagnosis of Alzheimer’s disease, the most common form of dementia, which significantly impacts survival rates among older adults. It introduced a pre-trained CNN, ResNet50, as an automatic feature extraction approach for analyzing MRI data. The performance of ResNet50 was evaluated against traditional classification methods, including Softmax, random forest (RF), and support vector machine (SVM). Results presented that the ResNet50 + Softmax approach achieved the highest accuracy, ranging from 85.7% to 99% on the ADNI dataset, outperforming SOTA models. This research highlighted the effectiveness of deep learning in automating the diagnosis process for Alzheimer’s disease.

The authors of^29^ concentrated on implementing a CNN from scratch to detect the stages of Alzheimer’s disease (AD) based on MRI images, addressing the need for early diagnosis to hinder disease progression. To enhance model accuracy and mitigate overfitting, data augmentation was applied to minority classes within the MRI dataset. Results demonstrated that the CNN achieved a remarkable accuracy rate of 99.1%, surpassing existing SOTA models. Additionally, the model displayed promising performance metrics, including 99% precision, sensitivity, and specificity, indicating its potential as a reliable clinical decision-support tool for diagnosing AD stages. Future work suggested enriching the dataset with MRI images from various medical sources to further validate and improve model performance.

The study in^30^ aimed to develop a DL model for the early diagnosis of AD utilizing MRI images, addressing the challenges of identifying early-stage AD, which is crucial for preventing disease progression. Utilizing two datasets including 6,400 and 6,330 images, the researchers employed a neural network with a VGG16. The results indicated high performance, achieving accuracy rates of 90.4% and 71.1%, along with AUC scores of 0.969 and 0.85 for the two datasets, respectively. The model outperformed existing SOTA approaches in identifying AD stages, including ND, VMD, MD, and MOD cases. This work had many advantages for instance, the model was trained on two different datasets. The findings highlighted the potential of DL techniques in improving early AD diagnosis and emphasized the importance of further research and dataset expansion to enhance diagnostic accuracy.

The survey in^31^ focused on the advancement of automated tools for the early detection and diagnosis of AD using DL techniques and neuroimaging modalities such as structural MRI (sMRI), PET, and fMRI. It highlighted the disadvantages of traditional handcrafted techniques and the need for transfer learning models to address the challenges of limited labeled datasets and high computational requirements. The paper reviewed various methodologies for AD detection, emphasizing the phases of image capture, pre-processing, feature extraction, and selection. It noted that sMRI was the preferred imaging modality and that data augmentation was underutilized despite its benefits. The VGG-16 architecture emerged as the most widely adopted transfer learning model, recognized for its simplicity and effectiveness in large-scale image recognition tasks. However, it was concluded that VGG-16 does not always outperform other transfer learning models. The analysis revealed that the current research faced challenges such as a lack of large annotated datasets and the opacity of deep learning models, which undermined trust among clinicians. The authors suggested future studies focus on creating comprehensive datasets, enhancing model transparency through explainable AI techniques, and developing user-friendly tools for seamless integration into clinical practice.

The study in^32^ addressed the challenge of early diagnosis of AD, highlighting the subtle biomarker changes that are often overlooked. It emphasized the potential of ML models, specifically SVM, in identifying individuals at risk for AD, while also noting the neglect of model explainability in current research. Utilizing a dataset from the National Alzheimer’s Coordinating Center (NACC) with 1,024 features and 169,408 records, the study successfully reduced the feature space and trained SVM models, which achieved impressive performance metrics: an F1 score of 98.9% for binary classification and 90.7% for multi-classes. In addition, the SVM model predicted AD progression over four years, attaining F1 scores of 88% and 72.8% for binary and multiclass tasks, respectively. To improve explainability, the study applied rule-extraction methods that generated understandable rules, validated with SHAP and LIME models. Key factors influencing AD risk, such as judgment, memory, ORIENT, and COMMUN, were identified, along with the importance of the Clinical Dementia Rating tool (CDRT) in classifying AD. The review highlighted the abundance of studies focused on detecting Alzheimer’s using images, noting that further enhancements and more precise techniques are still needed.

## The proposed methodology

A hybrid approach is developed to identify four types of Alzheimer’s disease utilizing a dataset of 6,400 images. This approach is based on DSR-GAN with a CNN model. The DSR-GAN is chosen for its high efficiency and capability to enhance image resolution, while the classification process is handled by the CNN^33^.

### The proposed DSR-GAN with CNN model

**Fig. 2:**
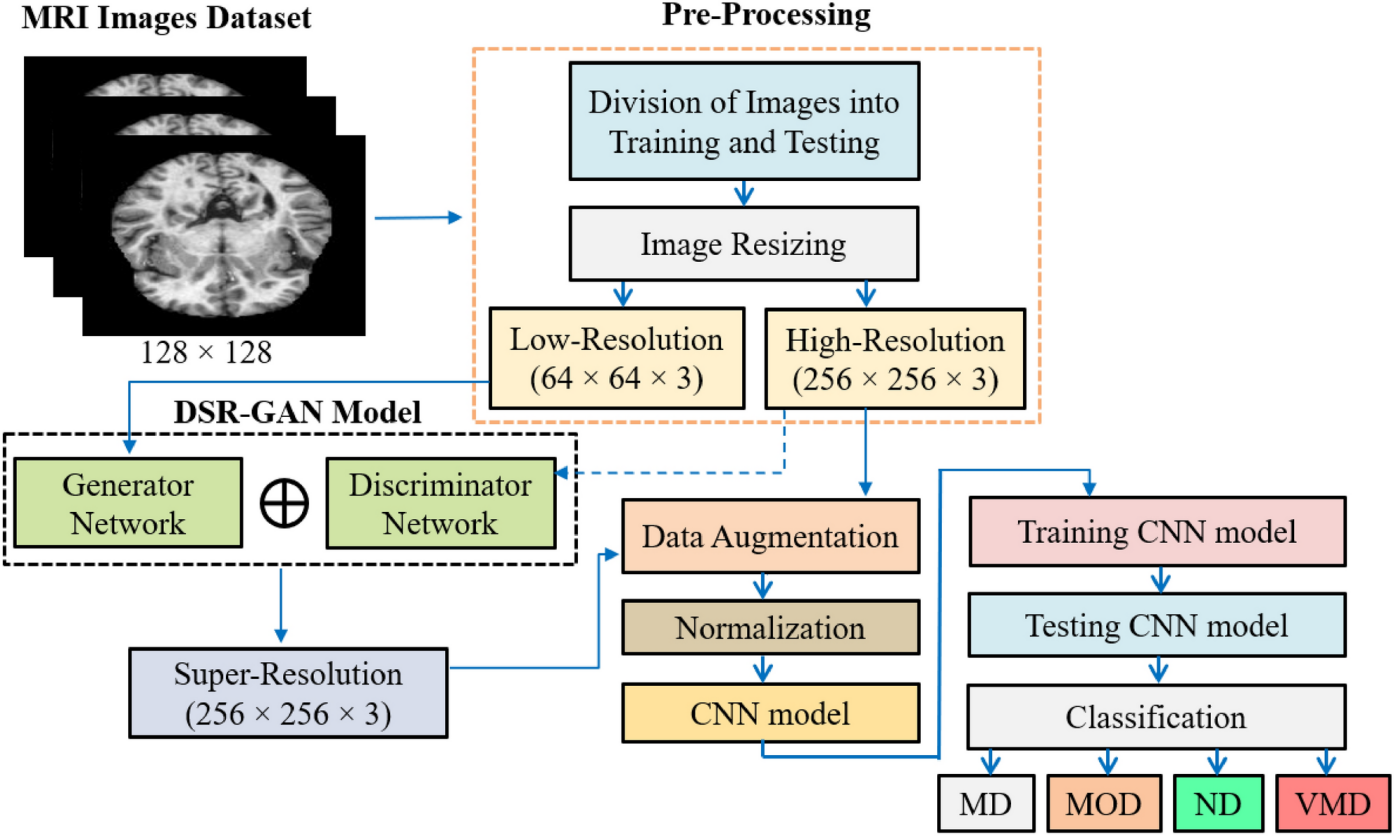
The workflow of the proposed hybrid approach.

In this study, a super-resolution (SR) technique is utilized on the selected Alzheimer’s disease images prior to classification with the CNN. The images are MRI’s four classes: “MD, MOD, ND, and VMD”. The original MRI images have dimensions 128 * 128 which are resized to 256 * 256 * 3 for high resolution and 64 * 64 * 3 for low resolution. Figure 2 illustrates the workflow of the proposed hybrid approach. The SR approach relies on a GAN algorithm to generate high-quality images. The DSR-GAN comprises two main components: the generator and discriminator networks.

**Fig. 3:**
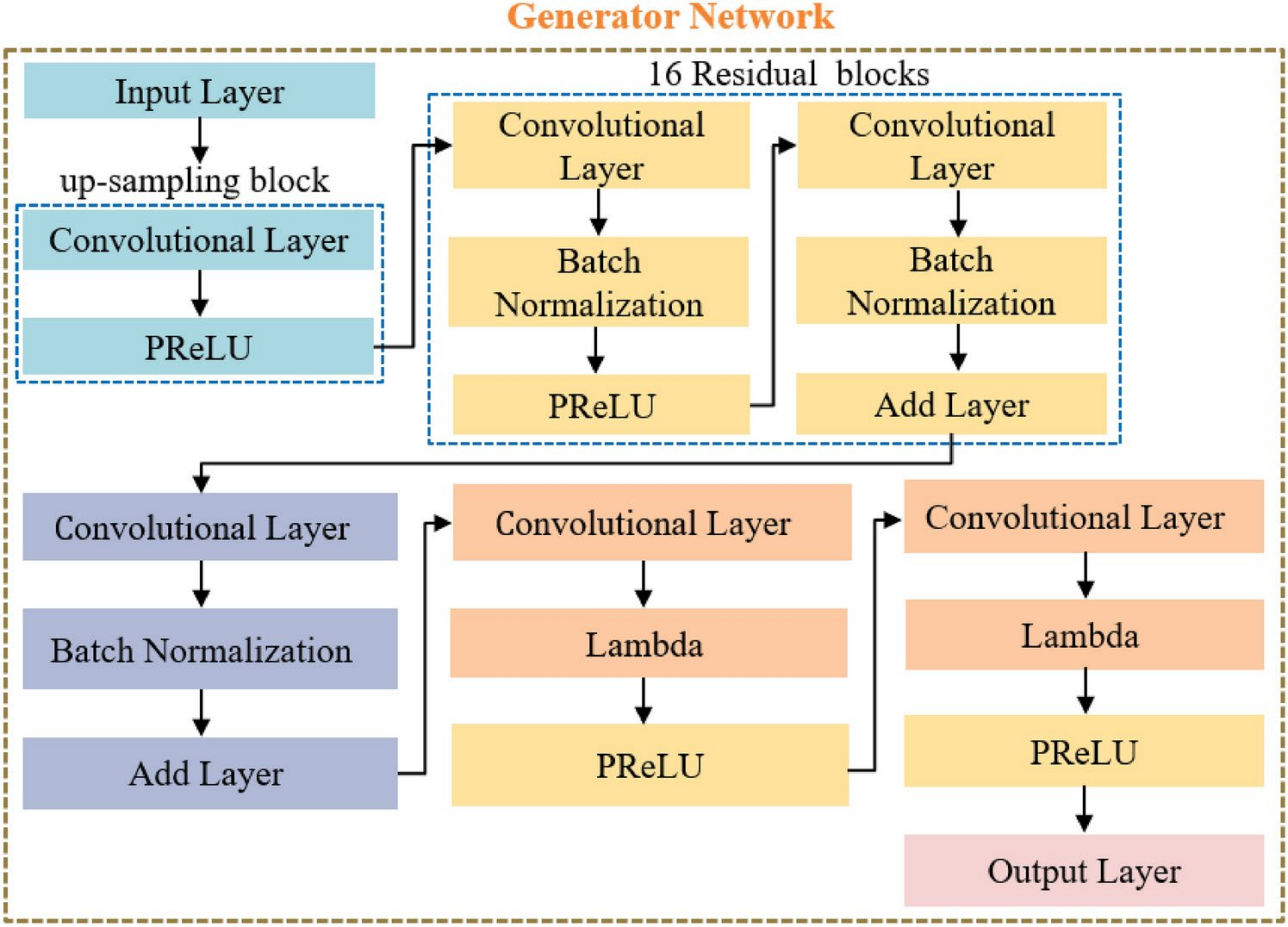
The structure of the generator network.

The structure of the implemented generator network is presented in Fig. 3. The generator features an input layer (IL) measuring 64 × 64 × 3 with a filter size of 3, followed by an up-sampling block that include a convolutional layer (CL) activated by a parametric rectified linear unit (PReLU) function. Residual blocks are repeated sixteen times, each comprising a CL, a batch normalization layer (BNL) then a PReLU, another CL, another BNL, and an Add layer (AL). Subsequently, three additional layers are included: CL, BNL, and AL, followed by two more layers: CL and Lambda, both activated by the PReLU function. Finally, CL as an output layer (OL) employs a sigmoidal function. Each CL includes 64 filters with 3 × 3 kernel sizes, resulting in a total of 1,550,659 learnable weights from the generator network using the super-resolution (SR) technique.

**Fig. 4:**
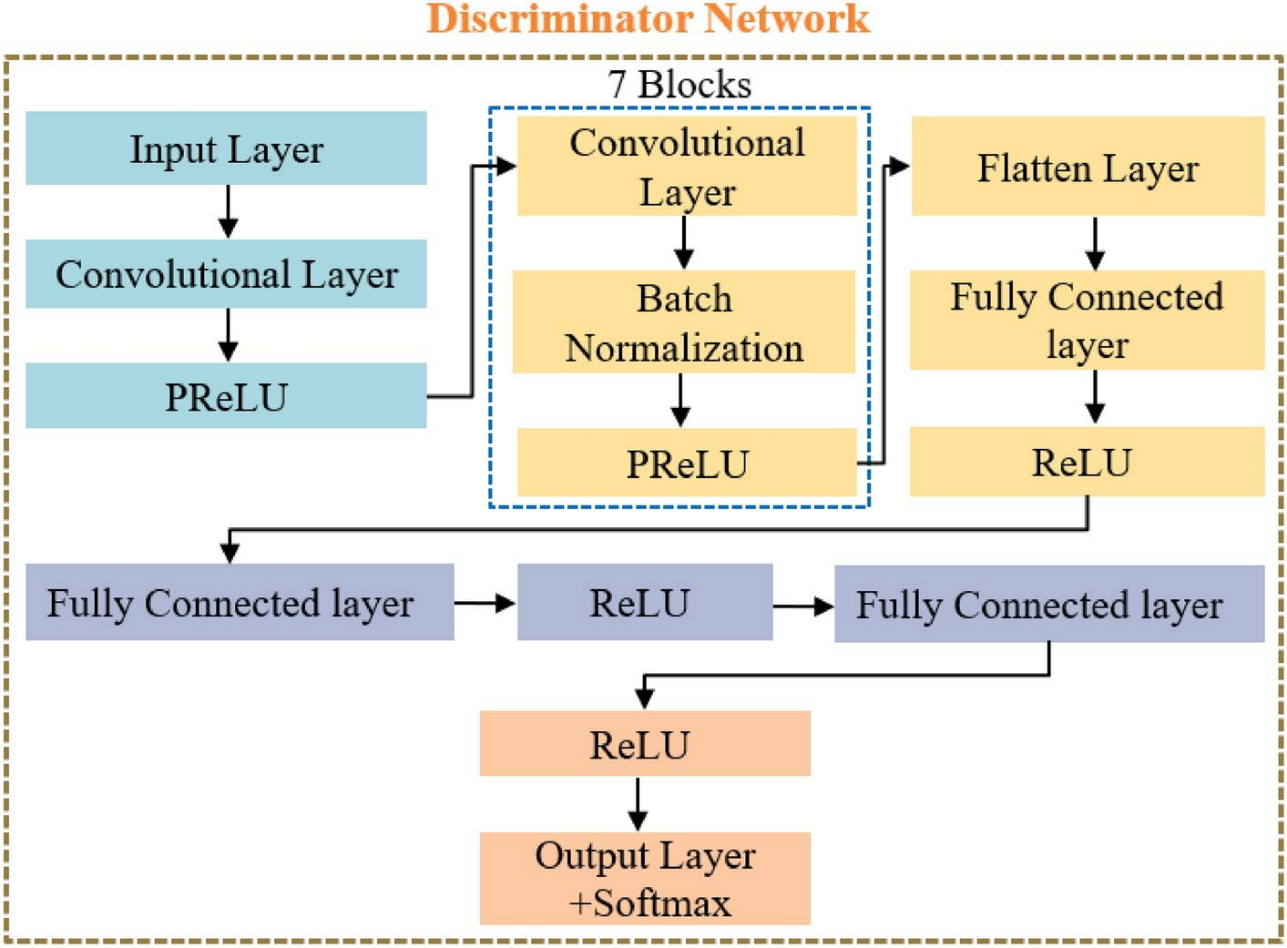
The structure of discriminator network.

Figure 4 shows the structure of the discrimiantor network. It includes an input layer (IL) and a convolutional layer (CL) with a ReLU activation function, then seven repeated frames, each containing a CL, a batch normalization layer (BNL), and activated by ReLU. This configuration is followed by a flattened layer (FL), then a fully connected layer (FCL), which uses a ReLU activation function which are repeated three times. Then a final dense output layer activated by a sigmoidal function. High-resolution (HR) images have dimensions of 256 × 256 × 3, while low-resolution (LR) images are derived from the HR images and measure 64 × 64 × 3. The scaling operator of 4 is used to determine the shape of the LR images from the HR images. For the SR technique, only 1,700 images are selected from a total of 6,400 images due to the extended training time required by the generator network. These images are normalized to values between 1.0 and -1.0. The generator consists of 109 layers while the discriminator includes 32 layers. The main goal of the discriminator network is to differentiate between genuine/original images and those that are generated/fake by the generator network.

**Fig. 5:**
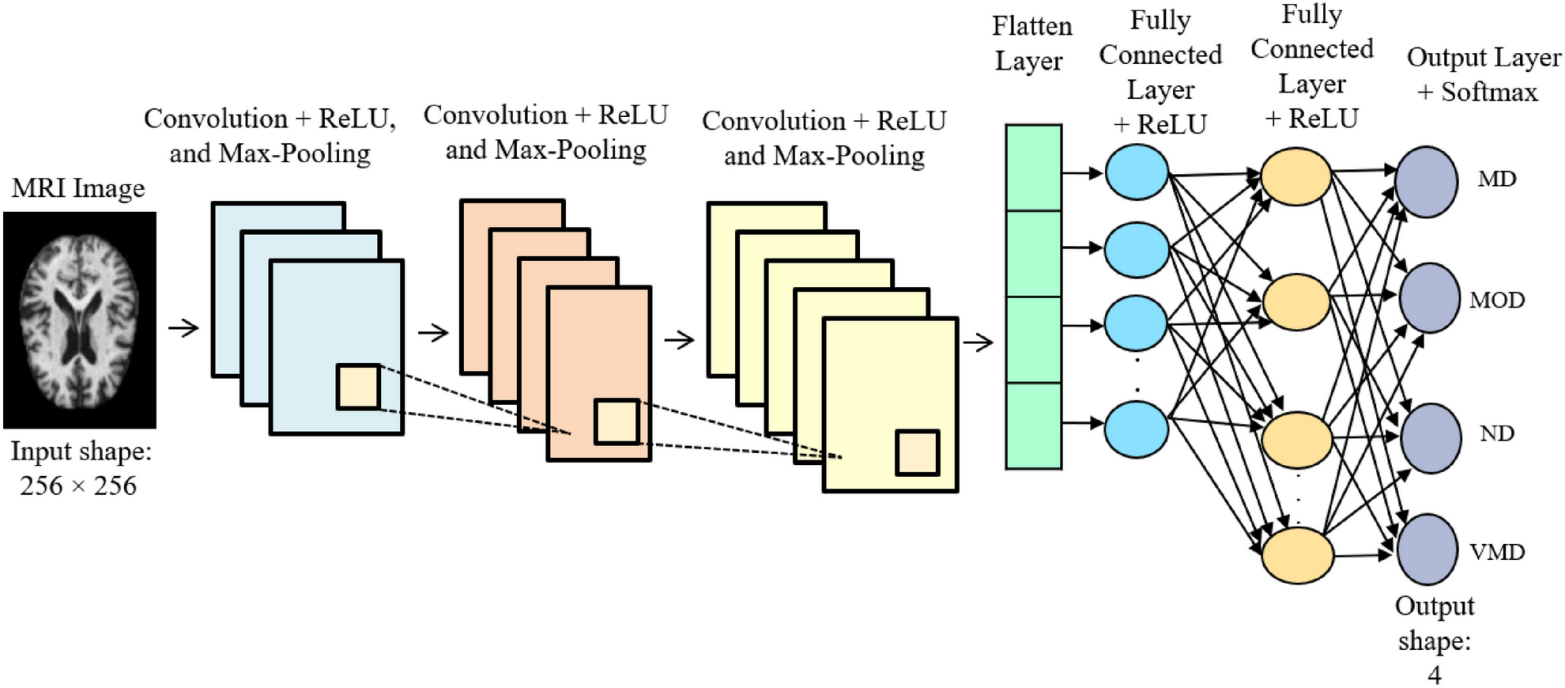
Architecture of the proposed CNN.

To evaluate the difference between the actual and predicted values, a categorical cross-entropy error function *E* is employed in the CNN, as shown in Eq. (1), where *y* represents the actual output, $\widehat{y}$ denotes the predicted outcome, and *N* indicates the number of labels. The optimization method is applied to adjust the weights, with a batch size set to 30 during the training phase of the CNN, which runs for 18 learning epochs at a speed of 0.0001. The total number of learnable weights in the DSR-GAN amounts to 86,746,434, whereas the CNN comprises 3,261,988 parameters. Figure 5 provides a detailed architecture of the layers utilized in the CNN model, where each input image has dimensions of 256 pixels in both height and width.

The CNN architecture consists of ten layers arranged in sequence. It starts with a convolutional layer activated by a ReLU function, which is followed by a max pooling layer to extract features from the dataset. This combination is repeated three times. Next, a flattening layer transforms the two-dimensional matrix into a one-dimensional vector. This is followed by two fully connected layers in cascade, concluding with an output layer that employs a softmax activation function. The applied hyperparameter settings for the CNN model: a batch size of 30 and number of epochs of 18.

1 $$E=-\frac{1}{N}{\sum }_{i=1}^{N}{\sum }_{k=1}^{K}\left[{y}_{k}^{\left(i\right)}{log}_{2}{\widehat{y}}_{k}^{\left(i\right)}+\left(1-{y}_{k}^{\left(i\right)}\right) {\mathit{log}}_{2}\left(1- {\widehat{y}}_{k}^{\left(i\right)}\right)\right]$$

### Hypotheses of the proposed work

The following hypotheses support the development of the hybrid DSR-GAN and CNN models for Alzheimer’s disease detection:

1. Hypothesis 1: The integration of a super-resolution (SR) technique with GAN will significantly enhance the clarity and detail of MRI images, improving the feature extraction capability of the CNN model.
2. Hypothesis 2: The optimized CNN model, when combined with high-quality images processed by the DSR-GAN, will outperform state-of-the-art models in classifying Alzheimer’s disease into four stages (Mild Dementia, Moderate Dementia, Non-Dementia, and Very Mild Dementia).
3. Hypothesis 3: Data augmentation techniques will mitigate overfitting and enhance the generalization of the CNN model, ensuring robust performance on unseen data.
4. Hypothesis 4: By optimizing hyperparameters, the proposed CNN model can achieve a near-perfect balance between accuracy, precision, recall, and F1-score, validating its efficiency for medical diagnostics.

### Dataset

**Fig. 6:**
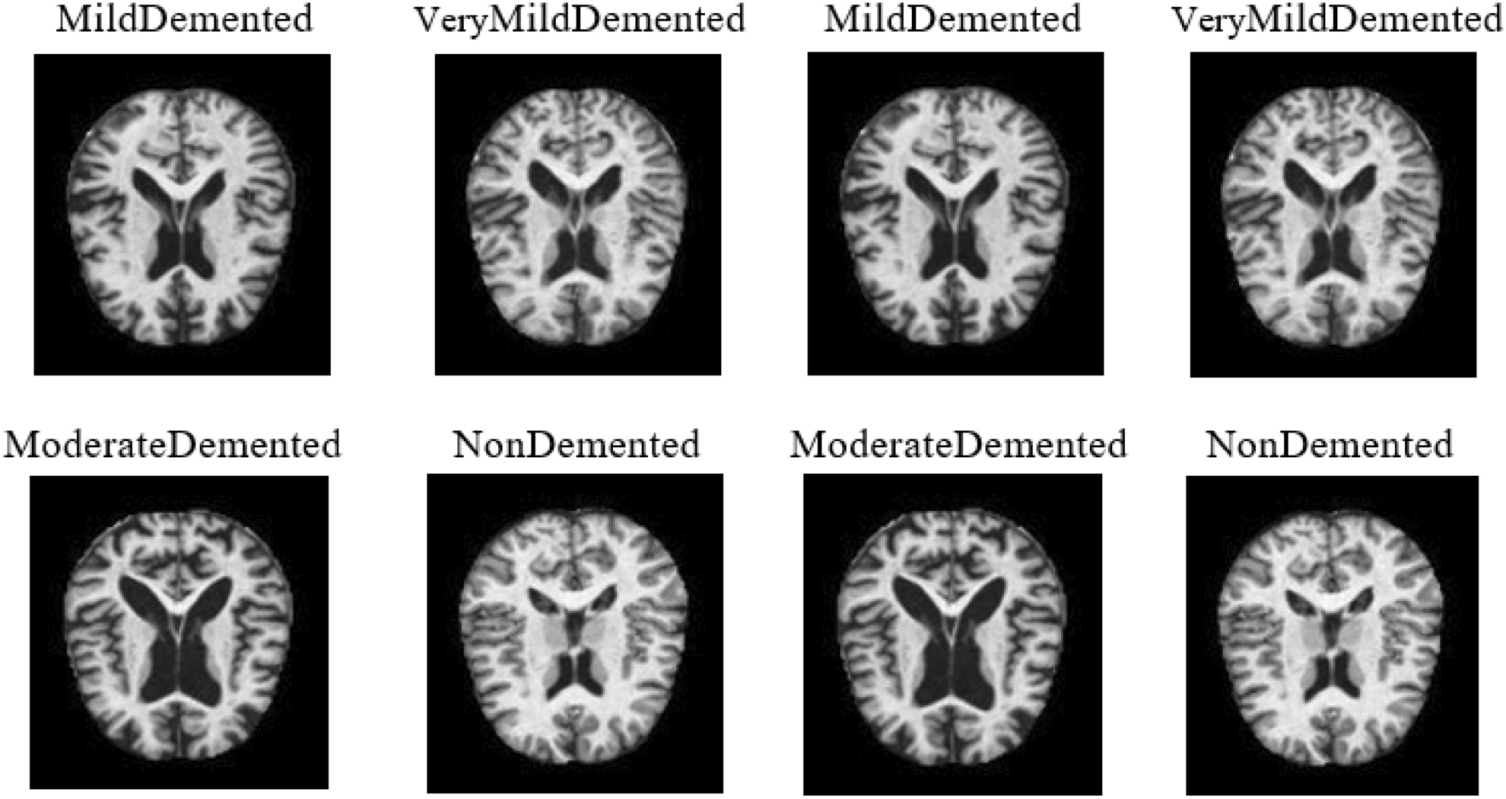
Some Samples of MRI images are utilized.

Figure 6 displays different MRI images sourced from the Kaggle website that were used for classification purposes^34^. The dataset comprises a total of 6,400 images across four categories: 896 for Mild Dementia (MD), 64 for Moderate Dementia (MOD), 3,200 for Non-Dementia (ND), and 2,240 for Very Mild Dementia (VMD). Each image is resized to dimensions of 256 × 256 pixels. Subsequently, a normalization procedure is performed on each image by rescaling the pixel values.

To mitigate overfitting in the DSR-GAN model, a data augmentation technique is utilized to enhance the dataset for the MD, MOD, ND, and VMD classes. This augmentation involves applying transformations such as width shifting, rotation, and height shifting. Specifically, each image is rotated by 10 degrees, shifted horizontally by 0.1, and shifted vertically by 0.1. Additionally, the total number of images in the dataset is tripled. Data augmentation techniques are also employed during the classification phase of the CNN. These techniques include horizontal flipping, rotation, and vertical flipping. Each image is rotated by 45 degrees and flipped either vertically or horizontally with a probability of 0.5.

The dataset is partitioned such that 70% allocated for training the DSR-GAN and CNN models, while 30% is set aside for testing and evaluating the performance of the suggested DL models.

### Assessing metrics

Several evaluation metrics are utilized to assess the efficiency of deep learning models^35^. Key metrics include the F1 score, accuracy, sensitivity, precision, and the area under the receiver operating characteristic curve (ROC). This area serves as a comparative tool to evaluate the precision of different classification deep learning models, highlighting their ability to accurately assess and differentiate MRI images related to AD. Additionally, AUC-ROC reflects the true positive rate (TPR), which indicates the proportion of MRI images correctly identified as containing AD out of the overall number of MRI images showing the condition. Conversely, the false positive rate (FPR) measures the proportion of images wrongly identified as having AD among those that do not exhibit the disease.

True positives (TPos.) refer to the count of observations correctly predicted as positive, while true negatives (TNeg.) indicate the number of observations accurately predicted as negative. False negatives (FNeg.) represent the number of diseased observations that were incorrectly classified, whereas false positives (FPos.) denote observations that were inaccurately identified as having Alzheimer’s disease, despite not being positive.

The testing accuracy (Acc.) of a specific deep learning model is calculated as the ratio of correctly predicted images to the overall number of images. This relationship is expressed in Eq. (2). Precision percentage (%Prec.) measures the proportion of MRI images correctly identified as belonging to a particular label, calculated as the number of true positives (TPos.) relative to the total number of detected images (TPos. + FPos.). Equation (3) outlines this calculation. Recall (Rec.) also referred to as sensitivity, represents the ratio of true positives (TPos.) to the overall number of actual positive images (FN + TPos.), as described in Eq. (4).

The F1 score (F1scr.) also known as the balanced F1 measure, is calculated as the harmonic mean of Precision and Recall, multiplied by a factor of 2. This relationship is outlined in Eq. (5). Consequently, the F1 score is generally considered a more reliable metric than the accuracy.

The AUC, or area under the ROC curve, is defined in Eq. (6). It ranges from 0 to 1, where higher AUC values indicate better performance of a deep learning algorithm across different image categories. Additionally, a deep learning model with a larger AUC demonstrates greater accuracy compared to one with a lower AUC.

2 $$Acc.=\frac{TNeg.+TPos.}{FNeg.+TNeg.+FPos.+TPos. }$$

3 $$Prec.=\frac{TPos.}{(FPos.+TPos.) }$$

4 $$Rec.=\frac{TPos.}{(FNeg.+TPos.) }$$

5 $$F1scr.=\frac{2\times Rec. \times Prec.}{[Rec.+Prec.] }$$

6 $$AUC={\int }_{0}^{1}TPR d(FPR)$$

To evaluate the effectiveness of the super-resolution (SR) technique implemented with GAN, the following metrics are employed: mean squared error (MSE), multi-scale structural similarity index measure (MS-SSIM), peak signal-to-noise ratio (PSNR), and structural similarity index measure (SSIM)^36–39^. These metrics are defined by Eqs. (7), (8), (9), and (10).

7 $$MSE=\frac{1}{n }{\sum }_{j=1}^{n}{\left({k}_{r }\left(j\right)-{i}_{y}\left(j\right)\right)}^{2}$$

8 $$PSNR=10 {log}_{10}\frac{{MAX}_{k}^{2}}{MSE }$$

9 $$SSIM = \frac{{\left( {2 \mu_{kr } \mu_{ky} + c_{1 } } \right)\left( {2{O^{\prime}}_{krky} + c_{2} } \right) }}{{\left( { \mu_{kr}^{2} + \mu_{ky}^{2} + c_{1 } } \right)\left( {{O^{\prime}}_{kr}^{2} + {O^{\prime}}_{ky}^{2} + c_{2} } \right) }}$$

10 $$MS-SSIM =\frac{1}{nm } {\sum }_{p=0}^{n-1} {\sum }_{j=0}^{m-1}SSIM$$

## Experimental results

**Fig. 7:**
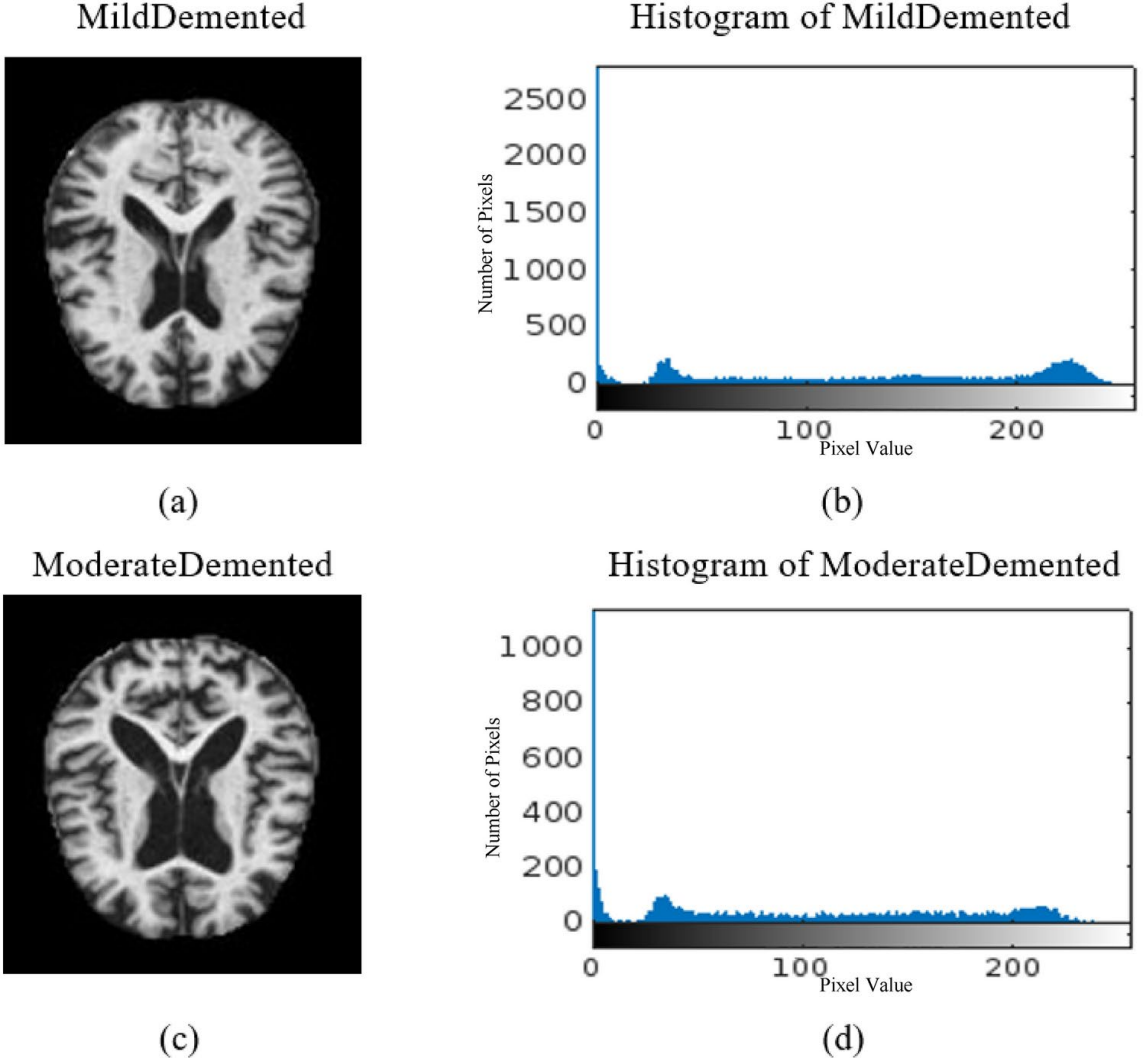
(**a**) MD image, (**b**) histogram of MD, (**c**) MOD image, (**d**) histogram of MOD.

**Fig. 8:**
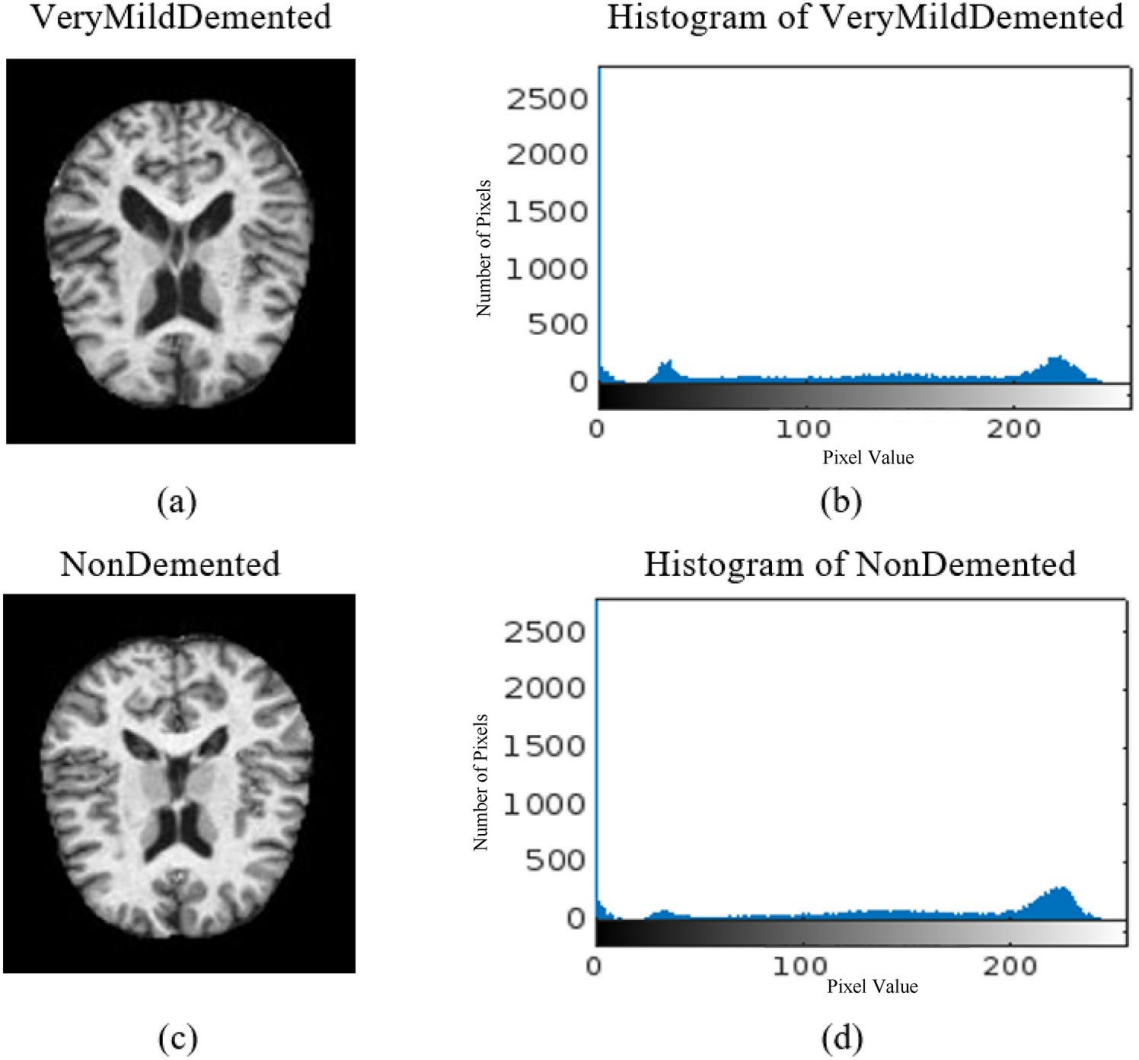
(**a**) VMD image, (**b**) histogram of VMD, (**c**) ND image, (**d**) histogram of ND.

The proposed DSR-GAN and CNN models are implemented using Python in Google Colab, which provides 25 GB of RAM and a P100 GPU. Figs 7 and 8 present four different samples with their histograms for images of Mild-Demented, Moderate-Demented, Non-Demented, and Very-Mild-Demented classes. Generally, the histogram is a valuable tool in medical image analysis for several reasons. It represents the distribution of pixel intensity values in an image, offering insights into its contrast, brightness, and content. There are several benefits of using a histogram for medical images: image quality analysis to evaluate its contrast and brightness. For example, in an MRI image, its histogram can help identify tissues with specific density or intensity ranges, aiding in detecting abnormalities like tumors.

**Fig. 9:**
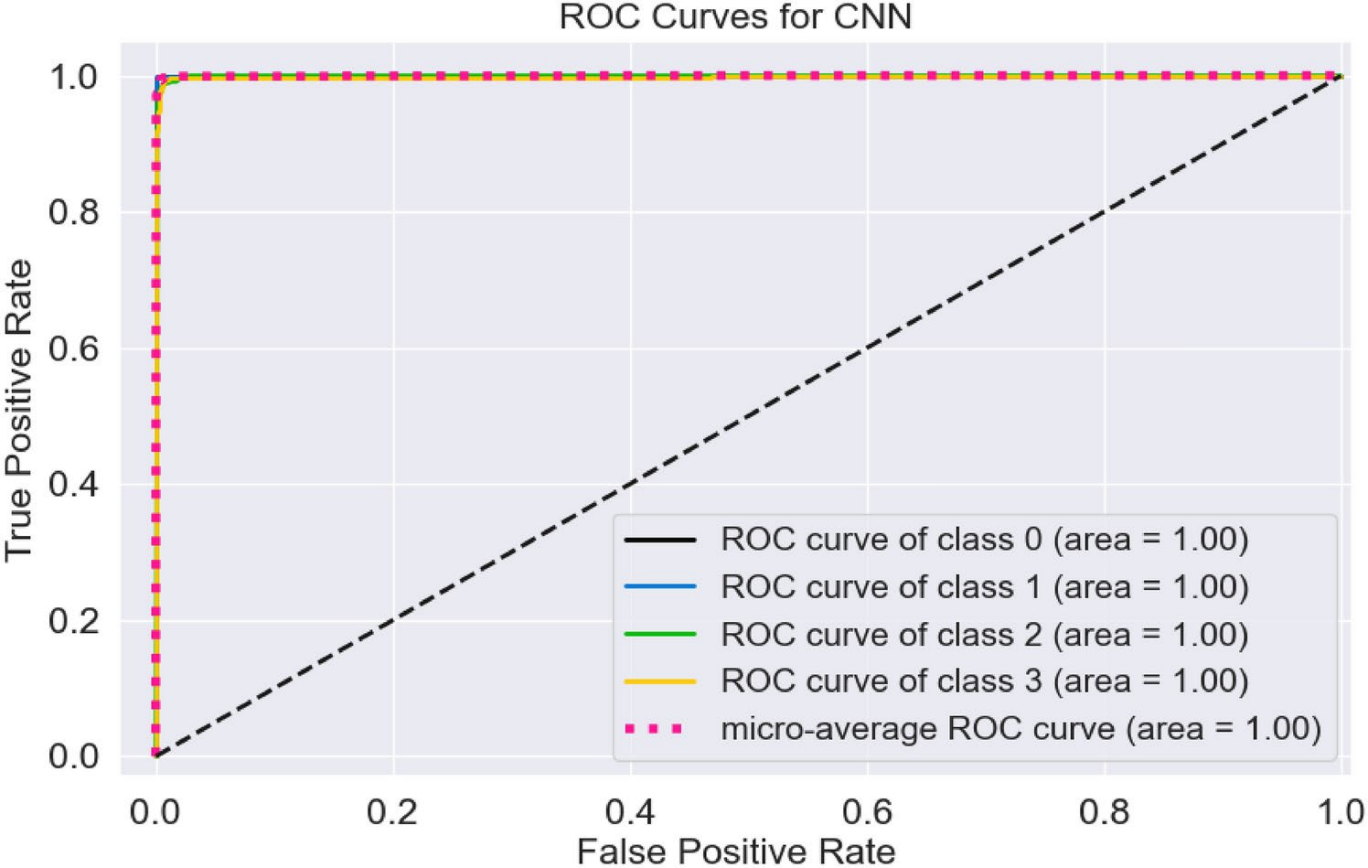
ROC curves for CNN.

The receiver operating characteristic curve (ROCC) is illustrated in Fig. 9. The ROC curves indicate TPR relative to the FPR levels. This Figure displays the ROC curves for the CNN, with all four classes achieving area values of 1.00, or 100%. The micro-average ROC area also reached 100%. These results indicate that the CNN performed flawlessly during the classification phase, with no errors detected.

**Fig. 10:**
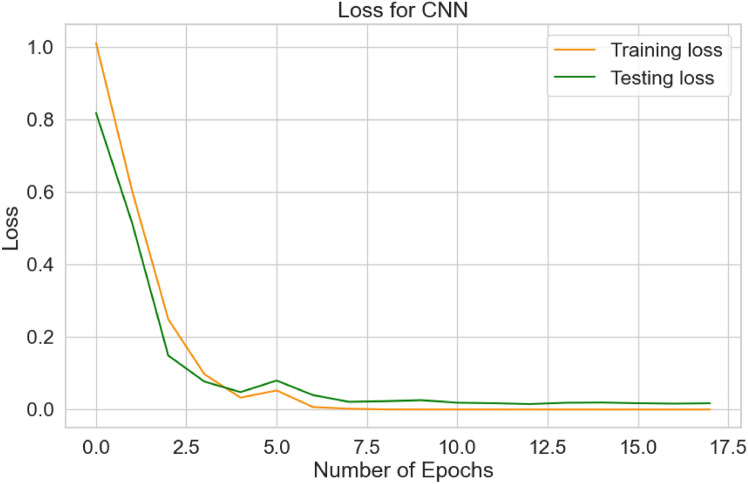
Loss curves for CNN.

**Fig. 11:**
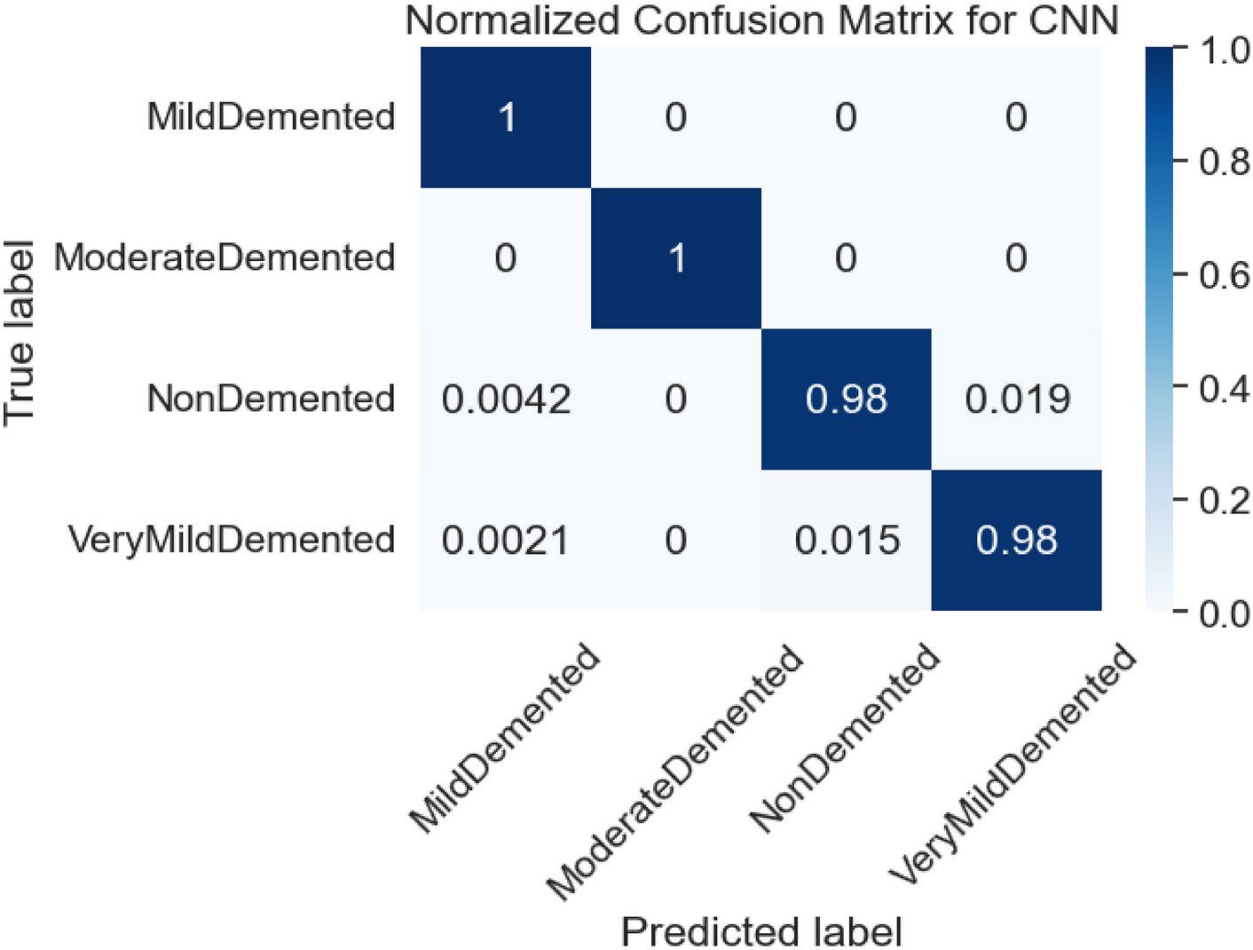
Normalized error matrix for CNN.

Figure 10 illustrates the error/loss curves of the CNN based on both training and testing images. As the number of training iterations increased, the error value decreased. The loss for the training dataset reached 0.00005 after 18 epochs, while the loss for the testing dataset was 0.0516. Figure 11 shows the normalized confusion matrix for the CNN, showing an accuracy of 1.0 (or 100%) for both the Mild-Demented (MD) and Moderate-Demented (MOD) classes. In contrast, accuracy for the Non-Demented (ND) and Very-Mild-Demented (VMD) classes is slightly lower at 0.98 (or 98%), referring to a small number of classification errors.

Table 1 provides an overview of three evaluation metrics for the CNN, derived from the test data used. The Macro and Weighted averages for the F1 score, Precision, and Recall across the MD, MOD, ND, and VMD classes are all recorded at 99.01%.

**Table.1:** Classification report for CNN.

Class	Precision	Recall	F1-score
MD	0.9938	1.0000	0.9969
MOD	1.0000	1.0000	1.0000
ND	0.9853	0.9771	0.9812
VMD	0.9813	0.9833	0.9823
Accuracy	-	-	0.9922
Macro average	0.9901	0.9901	0.9901
Weighted average	0.9901	0.9901	0.9901

**Fig. 12:**
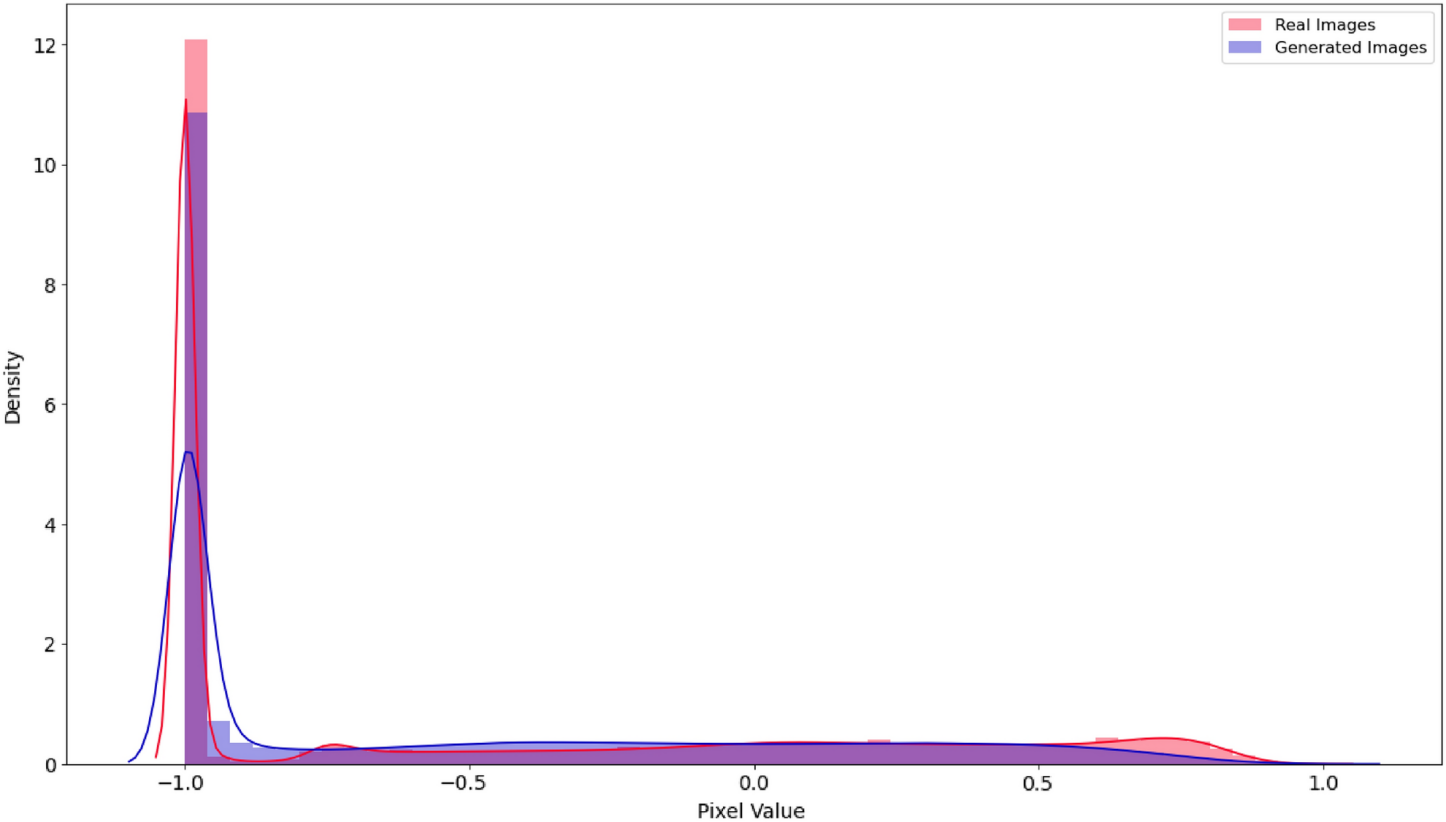
The relative frequency distribution for real and generated images.

**Fig. 13:**
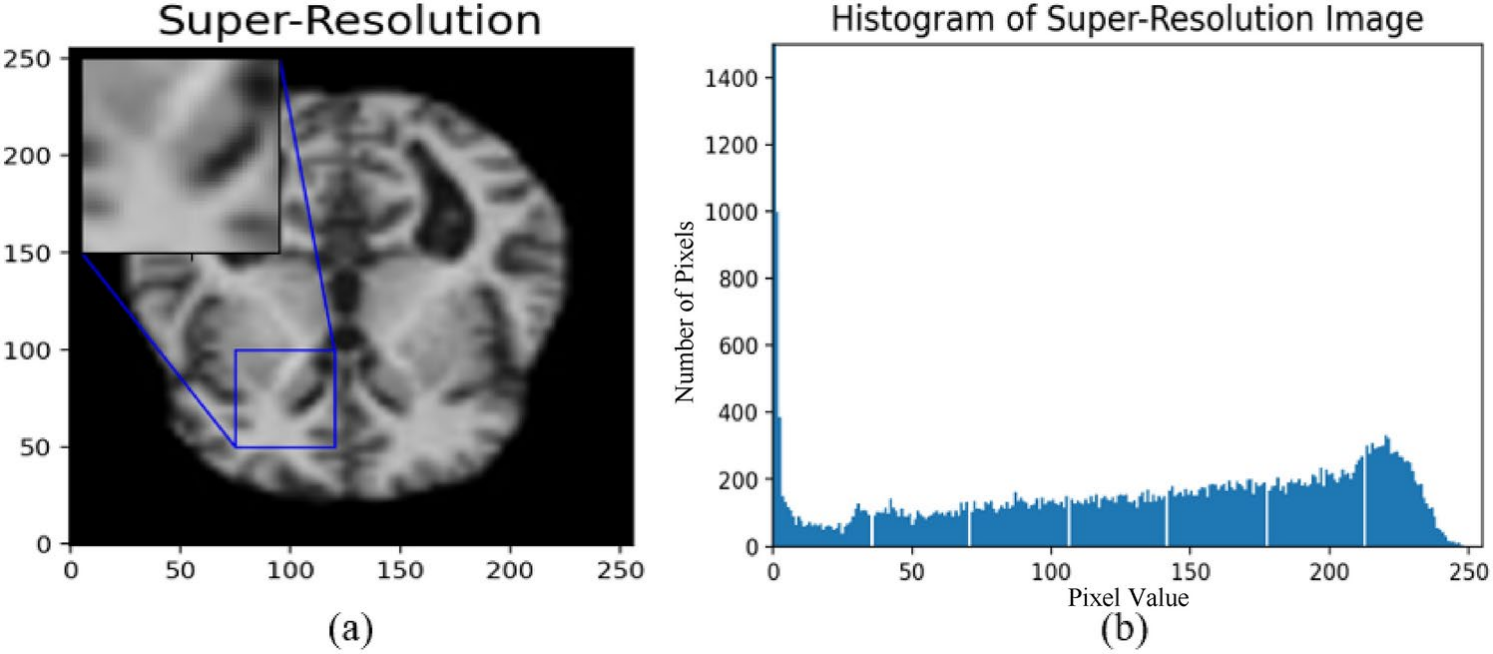
Results: (**a**) SR image; (**b**) Histogram.

Evaluating the GAN model qualitatively to test the generated sample plotting the distributions. In this test, we compare the generated images to the real samples by plotting their distributions. If the distributions overlap, that indicates the generated samples are very close to the real ones as in Fig. 12. The relative frequency distribution of a set of photographs is seen in this scatter plot. The highest frequency is shown by the red line, the lowest frequency by the blue line, and the lowest frequency by the purple line. The pixel value, which varies from -1.0 to 1.0, is represented by the x-axis. The density of images is shown on the y-axis. Figure 13 presents the outcome of the SR technique. Specifically, Fig. 11a depicts a super-resolution image. Meanwhile, Fig. 11b displays the histogram corresponding to this MRI image. A noticeable variation in the image resolution can be observed.

## Discussion

The findings indicate that the CNN model exhibits a low error rate and achieves high testing accuracy. The normalized confusion matrix and ROC curve further demonstrate that the CNN model effectively detects Alzheimer’s disease (AD).

The model’s performance is demonstrated in Fig. 10 to depict the model’s efficiency in relation to the loss level, achieving a value of 0.0516 for the CNN. Additionally, Fig. 11 displays the loss distribution across the four labels, indicating strong performance from CNN. Finally, Fig. 13 showcases the image resolution achieved during the super-resolution phase.

The peak performance achieved can be attributed to the meticulous fine-tuning of the CNN model’s hyperparameters, including the choice of optimizer algorithm, pooling size, error and activation functions, kernel dimensions, batch size, and the number of nodes in each layer, along with the specified number of learning epochs.

For the CNN model, when configured with 18 epochs and a batch size of 64 with softmax activation along with max-pooling and convolutional layer dimensions of 2 × 2 and 3 × 3 kernels, respectively, the testing accuracy (TA) achieved 96.89%. Conversely, adjusting the batch size to 32 and increasing the learning epochs to 40, while employing sigmoidal activation with max-pooling and convolutional layer dimensions of 3 × 3 and 5 × 5 filters, respectively, resulted in a TA of 98.78%. These findings indicate that hyperparameter settings play a vital role in enhancing model performance.

Table 2 showcases a comparison of the testing accuracy achieved by the suggested work against previously published techniques. Notably, the accuracy of the DSR-GAN with CNN surpasses the accuracy levels reported in references^19–28,30,32^.

**Table 2:** Comparison of the accuracy for the proposed work versus previous techniques.

Reference	Technique	Testing accuracy (%)
[^19^, 2022]	TL	91.7
[^20^, 2023]	Ensemble	95.89
[^21^, 2021]	DL	95.23
[^22^, 2022]	CNN	96.12
[^23^, 2022]	ADD	98.63
[^24^, 2022]	DNN	93.31
[^25^, 2019]	LSTM	93.35
[^26^, 2019]	CNN	90.05
[^27^, 2023]	CNN	99.05
[^28^, 2022]	CNN	92
[^29^, 2024]	ANN, VGG16	90.4
[^30^, 2024]	SVM	90.7
The proposed work	DSR-GAN with CNN	99.22

Table 3 presents a comparison between this work and other related published studies in terms of the techniques, data used, key findings, evaluation metrics, advantages, weaknesses, and future directions. It is clear that the integration of super-resolution with classification differentiates the proposed approach, addressing image quality issues often ignored in prior works. Also, most published studies reported accuracy and AUC; however, the proposed work includes additional metrics such as PSNR, SSIM and MS-SSIM, which adds depth to the evaluation of image processing quality. The robustness of the proposed work includes applying data augmentation extensively, which improves the generalizability of the CNN model.

**Table 3:** Comparison between this work and previous studies.

Study	Techniques	Data used	Key findings	Evaluation metrics	Advantages	Weaknesses	Future directions
^19^	Transfer learning with MRI data, AlexNet	Small MRI datasets	Classified AD into four stages	Accuracy: 91.7% across four AD stages	Efficient for small datasets	Low accuracy compared to state-of-the-art	Fine-tuning CNN layers, exploring other architectures, testing larger datasets
^20^	Ensemble model (VGG-16, EfficientNet-B2)	Imbalanced MRI datasets	Outperformed single models in accuracy and AUC	Accuracy: 97.35%, AUC: 99.64%	Addressed class imbalance, robust classification	Limited to imbalanced datasets	Test with larger datasets
^21^	CNN-based model (DEMNET)	Kaggle, ADNI datasets	High performance in classifying AD stages	Accuracy: 95.23%, AUC: 97%, Kappa: 0.93	Class imbalance handled via SMOTE	Lower performance on larger datasets	Robustness testing on ADNI
^22^	CNN on sMRI images	ADNI dataset	High accuracy, reduced parameters	High accuracy, automated feature extraction	Parameter efficiency	Limited robustness due to dataset constraints	Incorporate patient history
^23^	ADD-Net, a deep CNN model	MRI scans	Outperformed DenseNet169, VGG19	Accuracy: 98.63%, AUC: 99.76%	Lightweight model	Limited scalability to other imaging modalities	Incorporate transfer learning
^24^	Hybrid models (CNN, Bidirectional LSTM)	DementiaBank transcripts	Significant hyperparameter optimization	Accuracy: 93.31%, significant hyperparameter optimization	Achieving significant hyperparameter optimization via GridSearch	Achieved low accuracy	Improve performance on larger datasets
^25^	LSTM networks	Patient data over time	Predicts AD progression	Outperformed existing approaches in predictions	Focusing on the temporal relationships in patient data	Reached medium testing accuracy	Highlighted temporal relationships
^26^	Ensemble CNNs, patch-based approach	ADNI, NRCD datasets	Competitive accuracy in AD diagnosis	Accuracy: 85.55% (ADNI), 90.05% (NRCD)	Providing an effective framework	Achieved low testing accuracy	Address overfitting, data scarcity
^27^	Deep CNN architecture	MRI datasets	Achieved high classification accuracy	Accuracy: 99.05% in classification	High classification performance	High complexity architecture 7,866,819 parameters	Refine preprocessing, multi-modal approaches
^28^	ResNet50, automatic feature extraction	ADNI dataset	Outperformed traditional methods	Accuracy: 85.7%—99%	Achieved high accuracy	Consume long training time	Automation of diagnosis
^29^	Custom CNN with data augmentation	MRI dataset	Addressed overfitting, high-accuracy	Accuracy: 99.1%, 99% precision, sensitivity, specificity	High classification performance	Dataset diversity and size were limited	Enrich the dataset with diverse sources
^30^	Neural network classifier with VGG16	Two MRI datasets	High performance in early AD diagnosis	Accuracy: 90.4% & 71.1%, AUC: 0.969 & 0.85	The model was trained on two different datasets	Achieved low accuracy	Expand dataset, improve accuracy
^31^	Review of deep learning techniques	Various neuroimaging modalities	Identified challenges and methodologies	Identified VGG-16 as a preferred model	effectiveness in large-scale image recognition tasks	Reached low accuracy compared to other pre-trained models	Create comprehensive datasets, enhance model transparency
^32^	SVM with rule-extraction methods	National Alzheimer’s Coordinating Center dataset	Achieved high F1 scores for classification	F1: 98.9% (binary), 90.7% (multiclass)	Explainability, robust multi-class performance	High computational costs for feature space reduction	Focus on model explainability
This work	A hybrid of DSR-GAN and CNN	6,400 MRI images based on four classes	Achieved high accuracy with a super-resolution technique as an image processing	Accuracy: 99.22%, Precision: 99.01%, Recall: 99.01%, F1-score: 99.01%, AUC: 100%, PSNR: 29.30 dB, SSIM: 0.847, MS-SSIM: 96.39%	Novel hybrid model, super-resolution enhancement	Only 1,700 images dataset for super-resolution training	Large dataset and additional deep learning methodologies, such as Vision Transformer (ViT)

### Limitations of the proposed work

In this paper, some limitations are solved through applying different preprocessing techniques such as extensive data augmentation and super-resolution for the utilized data. Also, we applied a proper fine-tuning for the hyper-parameters of the proposed DSR-GAN and CNN models to achieve higher performance/accuracy than the previous published works. However, still remaining few limitations will be addressed in future research work to significantly enhance the models’ robustness and adoption in real-world scenarios/products, these limitations are as follows:

Computational Complexity: The DSR-GAN model involves 86,746,434 learnable parameters, requiring significant computational resources for training. This could limit its deployment in resource-constrained environments, such as smaller clinics or hospitals.

Limited Dataset for Super-Resolution Training: Only 1,700 images from the dataset are used to train the DSR-GAN due to computational constraints of the utilized hardware, potentially limiting the model’s ability to generalize for diverse image quality enhancements.

Scalability: The model’s reliance on high-quality super-resolution images and computationally intensive processes may pose challenges for real-time scalability and deployment.

## Conclusion and future work

This paper proposes the development of the DSR-GAN integrated with CNN to classify patients with Mild Dementia, Moderate Dementia, Non-Dementia, and Very Mild Dementia using brain MRI scans. These models are trained on a dataset consisting of images representing each dementia category. Furthermore, a super-resolution (SR) technique is developed to improve image quality prior to the CNN classification phase. The SR technique depends on a GAN model and is assessed by different metrics such as MS-SSIM, PSNR, and SSIM, achieving results of 96.39% for MS-SSIM, 29.30 dB for PSNR, and 0.847 for SSIM. The testing accuracy of the CNN model is found to be 99.22%, with a testing error rate of 0.0516.

The normalized error matrix and the area under the ROC curves are reported for each category, allowing for an evaluation of the model’s performance. The CNN model achieved an F1 score, precision, and sensitivity of 99.01%. Additionally, the area under the ROC curve is 100% across all classes, and the normalized confusion matrix for the CNN, shows a testing accuracy of 1.0 (or 100%) for the four classes: MD, MOD, ND, and VMD.

The accuracy of the CNN model is greatly influenced by its hyperparameters, including the loss function, optimizer algorithm, number of training epochs, learning rate, and batch size. Achieving high performance is closely linked to the optimal configuration of these settings.

The proposed models aid physicians by facilitating the automatic and precise identification of patients with Alzheimer’s disease, making it a valuable asset for medical diagnostics. The results show that both the DSR-GAN and CNN models exhibit high efficiency in classifying Alzheimer’s disease. Furthermore, these models could be applied to other imaging scenarios to expedite diagnoses, providing significant advantages for both healthcare professionals and patients.

For future research, it is recommended to expand the alzheimer application with the proposed models to a huge dataset. So, one could apply the proposed models on external datasets like ADNI or NRCD is lacking, which may hinder the model’s generalizability across different populations and imaging protocols. Also, one clould apply a temporal analysis for the proposed method lacks to analyze longitudinal data (e.g., changes over time in a patient’s MRI scans), which is crucial for understanding the progression of Alzheimer’s disease.

Further, exploring the integration of additional deep learning techniques, such as Vision Transformer (ViT), alongside the current images. To cover different diagnostic modalities, one could incorporate data from other modalities (e.g., PET scans, fMRI) , which could provide complementary insights for a more comprehensive diagnostic approach. Implementing explainable AI methods (e.g., SHAP or LIME) to enhance the trust and applicability of the proposed model in clinical settings.

## Data Availability

The dataset generated during and/or analyzed during the current study are available in the [Kaggle] repository, https://www.kaggle.com/datasets/drsaeedmohsen/alzheimer-dataset.
